# Fibrous Demineralized Bone Matrix (DBM) Improves Bone Marrow Mononuclear Cell (BMC)-Supported Bone Healing in Large Femoral Bone Defects in Rats

**DOI:** 10.3390/cells10051249

**Published:** 2021-05-19

**Authors:** René D. Verboket, Tanja Irrle, Yannic Busche, Alexander Schaible, Katrin Schröder, Jan C. Brune, Ingo Marzi, Christoph Nau, Dirk Henrich

**Affiliations:** 1 Department of Trauma, Hand and Reconstructive Surgery, Goethe University Frankfurt, 60590 Frankfurt am Main, Germany; tanja.irrle@gmx.de (T.I.); yannic.busche@gmx.de (Y.B.); Alexanderschaiblex@gmail.com (A.S.); marzi@trauma.uni-frankfurt.de (I.M.); Christoph.Nau@kgu.de (C.N.); d.henrich@trauma.uni-frankfurt.de (D.H.); 2 Center of Physiology, Cardiovascular Physiology, Goethe University Frankfurt, 60590 Frankfurt am Main, Germany; schroeder@vrc.uni-frankfurt.de; 3 German Institute for Cell- and Tissue Replacement (DIZG, gemeinnützige GmbH), 12555 Berlin, Germany; j_brune@dizg.de

**Keywords:** critical-size defect, tissue engineering, BMNC

## Abstract

Regeneration of large bone defects is a major objective in trauma surgery. Bone marrow mononuclear cell (BMC)-supported bone healing was shown to be efficient after immobilization on a scaffold. We hypothesized that fibrous demineralized bone matrix (DBM) in various forms with BMCs is superior to granular DBM. A total of 65 male SD rats were assigned to five treatment groups: syngenic cancellous bone (SCB), fibrous demineralized bone matrix (f-DBM), fibrous demineralized bone matrix densely packed (f-DBM 120%), DBM granules (GDBM) and DBM granules 5% calcium phosphate (GDBM5%Ca2^+^). BMCs from donor rats were combined with different scaffolds and placed into 5 mm femoral bone defects. After 8 weeks, bone mineral density (BMD), biomechanical stability and histology were assessed. Similar biomechanical properties of f-DBM and SCB defects were observed. Similar bone and cartilage formation was found in all groups, but a significantly bigger residual defect size was found in GDBM. High bone healing scores were found in f-DBM (25) and SCB (25). The application of DBM in fiber form combined with the application of BMCs shows promising results comparable to the gold standard, syngenic cancellous bone. Denser packing of fibers or higher amount of calcium phosphate has no positive effect.

## 1. Introduction

Large bone defects occur after high force trauma, osteomyelitis or tumor resection. The large defects have always been a major challenge for treatment and costs. The gold standard is the transplantation of autologous or allogeneic bone into the bone defect. This therapy is unfortunately plagued with different kinds of problems. There is a limited availability of autologous bone, the surgical times are extended, loosening of the alloplastic implant is possible in the course of treatment and there are also possible immunogenic reactions to allogenic grafts [1,2,3]. Due to these limitations, it is necessary to find an appropriate substitute. Many different materials, from human allografts to xenografts and artificial bone materials, are available on the market. All these materials were tested alone or in combination with cells in different bone defect models [4,5].

In recent times, the use of osteoinductive cells combined with an osteoconductive scaffold has been proved to lead to better bone healing. A significant increase in vascularization by endothelial progenitor cells (EPCs) [6,7] and a bone mass increase assisted by mesenchymal stem cells (MSCs) in critical-size defects of the femur [6,8] and of the skull [9] were shown in rats. However, MSCs/EPCs are subject to certain drawbacks. They must be culture-expanded prior to use, which might increase the risk for genetic alterations [10] or contamination with pathogens. Additionally, it takes time to prepare the cells, and the costs are very high. Moreover, they are difficult to reconcile with clinical reality. Once the cells have been harvested, the patient must wait 3 weeks for cultivation before implantation. Through these disadvantages, the idea of the use of bone marrow mononuclear cells (BMCs) containing (immature) lymphocytes, (immature) monocytes and progenitor cell populations has been developed for the treatment of large bone defects [11,12,13]. BMCs can be isolated and transplanted into the bone defect within 24–36 h, thereby being well tolerated. Furthermore, in a clinical phase I study in patients with proximal humerus fracture, the good tolerability of the local BMC application was demonstrated [12]. It has been shown that BMCs contain several cellular subsets with proven regenerative potential such as (immature) monocytes, hematopoietic stem cells (HSCs), a putative source of EPCs and precursors of MSCs [14,15,16] and support mechanically stable bone healing in a dose-dependent manner [17,18,19,20]. A highly BMC-compatible [11] and well-tolerated bone substitute [21] is demineralized bone matrix (DBM). DBM is produced from human cortical bone and offers a porosity equal to natural human bone micropores with a diameter of 1–2 µm. DBM particles are sterilized by a validated peracetic acid-based process (data published by the manufacturer).

It has been repeatedly demonstrated that the surface/volume ratio of the bone graft substitute material can be a decisive factor in the healing process [22,23]. The fabrication of fibrous bone replacement material leads to a bigger surface and a higher binding capacity of cells which might further improve bone healing [24]. A possible disadvantage of the fibers is a high compression inside the fracture site leading to hypoxia and subsequent death of the seeded regenerative cells. One solution to this problem could be to mix in granular bone fragments, which locally vary the packing density of the fibers, thus minimizing compression and possibly stabilizing the fibers in the defect. In addition to the shaping of the bone replacement material, the availability of bioactive ions in the bone replacement material, such as calcium ions, is another significant aspect in supporting bone healing. Calcium ions can induce the expression of the bone growth factor bone morphogenetic protein 2 (BMP2) [25,26,27] and enhance differentiation and survival of EPCs, as well as induce early vascularization in critical-sized bone defects [28,29]. Regarding the osteogenic potential, it is known that DBM contains osteogenic proteins; therefore, it can be assumed that the access to and the release of these factors are favored by the fibrous form, and thus there is an increased osteogenic potential for fibrous DBM compared to the granulated version [30]. However, the process of demineralization leads to extensive depletion of osteoinductive ions such as calcium from DBM, and thus a higher amount of calcium might improve DBM’s osteoinductive capacity.

Keeping this in mind, we hypothesized (1) that fibrous DBM supplemented with granular DBM and BMCs is superior to granular DBM and BMCs alone. Furthermore, it was hypothesized (2) that fibrous DBM with 5% (*w*/*w*) residual calcium phosphate and BMCs improves bone healing compared to fibrous DBM without residual calcium but seeded with BMCs. Both hypotheses were investigated in our established rat 5 mm femur defect model.

## 2. Materials and Methods

### 2.1. Ethics and Animal Care

All animal experiments were performed in accordance with regulations set forth by our institution’s animal care and oversight committee (Project No. FK/1075; Regierungspräsidium, Darmstadt, Germany) in accordance with German law. Sixty-five 8–10-week-old male Sprague Dawley rats (Janvier Labs, Saint Berthevin, France) weighing approximately 250–300 g were housed in cages with three to four animals per cage. Housing conditions were identical for all rats; they were kept in rooms in which temperature (15–21 °C), airflow and light (14 h day and 10 h night) were controlled. Rats were given access to rat chow and water *ad libitum*. Nine days of acclimation of the animals before surgery were given. Animal wellbeing was ascertained by veterinarians daily during the first week after surgery and weekly thereafter.

### 2.2. Preparation of Fibrous Scaffold

Tissue grafts in Germany are regulated as medicinal products under the German Medicinal Products Act (Arzneimittelgesetz, AMG). All tissues were acquired from nonprofit tissue donation organizations after informed consent and according to German AMG and TPG (Transplantation Act, Transplantationsgesetz) regulations. Stringent serological screening was then followed by tissue preparation under controlled computerized numerical control (CNC) mill conditions, resulting in a cortical and a cancellous bone fraction. Subsequently, the tissue was partially demineralized and subjected to a validated sterilization process as described previously [31]. The fibrous tissue scaffold was then preserved by lyophilization and stored for later use.

### 2.3. Scaffolds

The animals were randomly divided into five test groups. They received different DBM preparations supplemented with syngenic BMCs isolated from donor rats, and in one control group, bone defects were filled solely with syngenic cancellous bone (SCB) obtained from donor animals, which matches the current gold standard. Bone defects in the control group were filled with SCB without additional BMCs. In the second group, the femoral defects of the animals were filled with a combination of demineralized cancellous bone and demineralized fibers in the relation 25% to 75% (f-DBM). In the third group, the same material as in the first group was used with the exception that 120% of the material was used to reach a denser packing (f-DBM 120%). For the normally packed groups, 100 mg of fibers was combined with 33.3 mg cancellous bone. In the 120% group, respectively 120 mg fibers was combined with 40 mg cancellous bone. The hydration of the material and the loading with BMCs were performed in one step by incubation of the material with 100 µL cell suspension containing 28.2 × 10^4^ BMCs over 10 min. In the fourth group, DBM granules (GDBM) were used; in the fifth group, DBM granules with 5% residual calcium (Ca2^+^) phosphate were implanted. The granular DBM (German Institute for Cell and Tissue Replacement (DIZG gemeinnützige GmbH, Berlin, Germany)) used was sterilized using a validated, GMP-conformable process and approved as a medicinal product under §21 of the German Medicinal Products Act (license number: PEI.H.03358.01.1). Fibrous DBM was prepared under controlled computerized numerical control (CNC) mill conditions; the bone was clamped into the CNC machine and cut into fibrous tissue scaffold by a milling head, resulting in a cortical and a cancellous bone fraction. Subsequently, the tissue was partially demineralized and subjected to a validated sterilization process [31]. All DBM was produced from bone of serologically screened donors by validated procedures, including decellularization, sterilization and preservation [31] (Table 1).

### 2.4. BMC Isolation and Seeding

**Figure 1:**
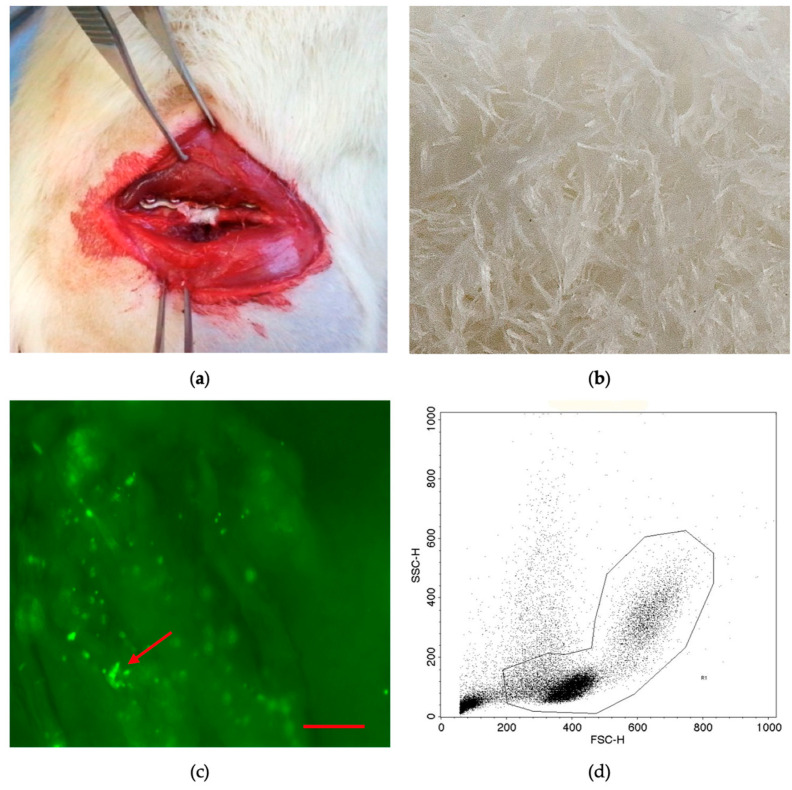
Plate-stabilized and filled femoral defect (**a**). A 5 mm critical-size defect in the right femur of a rat is stabilized with a 5-hole plate. The gap is filled with demineralized bone matrix fibers (f-DBM) loaded with BMCs. Macroscopic image of the fibrous tissue scaffold (**b**). Fluorescence microscopic image; adherent CFSE-stained BMCs (green dots, marked with red arrow) can be seen at different depth levels of the f-DBM plexus (original magnification 50×) (**c**). FACS characterization of rat BMCs (**d**). Mainly mononuclear cell types (lymphoid and monocytic cells) remain after density gradient centrifugation.

Rat BMCs were isolated from femora and tibiae of donor animals. The bones were removed aseptically. The proximal and distal parts of the bone were cut off. Bone marrow was rinsed out of the bone using a sterile syringe and phosphate-buffered saline (PBS). The bone marrow was diluted with PBS (1:3), and mononuclear cells were isolated by density gradient centrifugation with Ficoll (1.077 g/cm^3^, Biochrom, Berlin, Germany) at 800 *g* for 20 min without break at room temperature [18]. Afterward, mononuclear cells were washed twice with 25 mL PBS (800 g) and counted. To ensure that the BMC preparation contained primarily mononucleated cells, FACS analysis (FACScalibur, BD Biosciences, Heidelberg, Germany) was performed. BMCs were identified by their forward scatter and side scatter characteristics (Figure 1).

The preweighted scaffolds were placed in individual wells (area = 2 cm^2^) of a 24-well plate (Nunc, Wiesbaden, Germany) using sterile forceps. A cell suspension of 28.2 × 10^4^ BMCs [20] in a volume of 100 μL was dripped slowly on the scaffolds and incubated for 5 min at 37 °C. After incubation, the cell suspension not absorbed by the scaffold was removed and dripped over the material once again and incubated for a further 5 min. A parallel setup using CFSE-prestained BMCs was used to confirm adhesion to the material using fluorescence microscopy (Figure 1). CFSE staining (Thermo Fisher, Waltham, MS, USA) was performed following the guidelines of the manufacturer.

### 2.5. Surgical Procedure

A general anesthesia with 2 mL of a mixture of Ketavet (70 mg/kg) and Rompun (10 mg/kg) was given intraperitoneally. Under aseptic conditions, the right rat femur was dissected. A five-hole plate was prepared (Miniplate–Locking Plate LCP Compact Hand 1.5 Straight, DePuy-Synthes, Dubendorf, Switzerland) and positioned on the femur and secured in place with four 1.3 mm cortical screws (Compact Hand, DePuy-Synthes, Dubendorf, Switzerland), leaving the middle hole free. Using a Gigli saw (RI-Systems, Landquart, Switzerland) a 5 mm defect of the femur under the free middle hole of the plate was created. In a previous study, a 5 mm defect in a rat femur was shown to be a critical-size defect [32]. The different scaffolds were implanted into the segmental defects, and the wound was closed in two layers with continuous subcutaneous stitches using a 4/0-monofilament nylon suture. Postoperative analgesia was performed with 2.6 mg/kg carprofen s.c. directly after surgery and the first following day. Over the following five days, 2.5 mg/100 mL tramadol was provided in the drinking water as pain treatment. The rats were housed for eight weeks under standard conditions and nutrition.

The animals were sacrificed after eight weeks by inhalation anesthesia and following intracardial pentobarbital injection (500 mg/kg).

### 2.6. Biomechanical Characterisation

The bones were stored after plate removal at −80 °C until mechanical testing. Biomechanical properties of the femur’s defect site were measured by a destructive three-point bending procedure using a material testing machine (Zwicki-line Z5.0, Zwick-Roell, Ulm, Germany). Bending until failure was performed by lowering a bar onto the femur, using a constant deflection speed of 0.1 mm/s. Load and deflection were recorded continuously. The ultimate load (slope of the elastic deformation part of the load/deformation curve) was then calculated using the Testexpert-II software (Zwick-Roell, Ulm, Germany) in relation to the also measured matching contralateral femur.

### 2.7. Histological Assessment

Callus formation and bone maturation were assessed by means of histomorphometric analysis of decalcified sections taken from the bone defect and stained with Movat pentachrome. For histological staining, bones were carefully defrosted and fixed in Zinc-Formal-Fixx, 10%, over 20 h (Thermo Electron, Pittsburgh, USA) followed by decalcification for 14 days in 0.25 M Trizma base (Sigma-Aldrich, Taufkirchen, Germany) and 10% EDTA (Sigma-Aldrich, Taufkirchen, Germany), pH 7.4. Decalcified bones were embedded in paraffin; longitudinal sections (3 μm) were taken. Movat pentachrome staining of paraffin-embedded histological slides was performed as published by Garvey et al. [33] using a staining kit according to the manufacturer’s instructions (Morphisto, Frankfurt, Germany). New bone formation and cartilaginous tissue area were then analyzed in the defect site using the software ImageJ (https://imagej.nih.gov/ij/, accessed on 17 December 2020), and the relative tissue positive area of the entire defect zone was calculated.

Vascularization was measured with immunostaining of α-smooth muscle actin (α-SMA). The sections were incubated with monoclonal mouse anti-rat α-SMA (1 h at room temperature, final concentration 2 μg/mL, clone 1A4, antibody-ID: AB_262054, Abcam, Cambridge, UK). As secondary antibody, a polyclonal HRP-conjugated anti-mouse IgG (Simple Stain Rat MAX PO, Nichirei, Tokyo, Japan) was applied for 30 min, followed by incubation with 3-amino-9-ethylcarbazole (AEC, Sigma-Aldrich). Finally, a counterstain with hematoxylin was performed. In order to prevent the misinterpretation of α-SMA-positive cells as blood vessels, α-SMA-positive events below 10 μm in diameter were excluded [34].

All slides were analyzed using light microscopy. High-resolution images depicting the whole defect zone in each case were created by automated stitching of multiple single frames covering the whole defect using the software BZII Analyzer (Keyence, Neu-Isenburg, Germany). Two independent observers blinded to the group setup analyzed the samples.

Bone healing score was determined analogously to Han et al. [35]. The remnant defect was defined as defect area minus newly formed bone tissue. Higher values indicate better bone healing.

### 2.8. µCT-Analysis

To assess bone mineral density and callus volume, μCT analysis was performed with a high-resolution in vivo micro-CT Skyscan 1176 (Bruker AXS, Karlsruhe, Germany). The long axis of the femur was lined up orthogonally to the axis of the X-ray beam (Al 0.5 mm; voltage: 50 kV; current: 500 μA; frame average: 7; rotation ra.: 180; rotation st.: 0.5), and the region of interest was placed on the defect. Isotropic voxel size was 18 μm^3^. Two-dimensional CT images were scanned, reconstructed using a standard back convolution procedure and saved in 3D arrays. Bone mineral density (g/cm^3^) in the bone defect was calculated from the µCT data using a phantom with known density. For total volume and bone volume, measurement data were treated using a global fixed threshold (60 to 240 grey levels) with the same volume of interest. A virtual cylinder was centered in the middle of the defect with a length equal to 5 mm, and total volume and bone volume were calculated with Image J (https://imagej.nih.gov/ij/notes.html, accessed on 17 December 2020). The volume of newly formed bone or scaffold could not be distinguished from the implanted bone, similar to earlier studies [36]; thus, the total volume and the bone volume were assessed.

### 2.9. Statistics

Results are presented as box plots of the median in diagrams or as mean and standard deviation in the description of the results. A nonparametric Kruskal–Wallis test with Bonferroni–Holm-corrected Conover–Iman post hoc analysis was used for comparisons between the groups using the statistical software Bias 11.10 (Epsilon-Verlag, Darmstadt, Germany. *p* values < 0.05 indicate statistical significance. *p* values between 0.05 and 0.1 were rated as statistical trend.

## 3. Results

### 3.1. Animal Care/Complications

In total, four animals were excluded. Three animals were excluded due to pin loosening, and one animal died due to breathing arrest during surgery. The numbers of excluded animals per group were one in f-DBM (120%), one in the GDBM group and one in the SCB group. No macroscopically visible side effects of the f-DBM were recorded. The product was considered to be safe to use in prior experiments [21].

### 3.2. Similar Biomechanical Properties of Bone Defects Treated with f-DBM and Syngenic Cancellous Bone 

**Figure 2:**
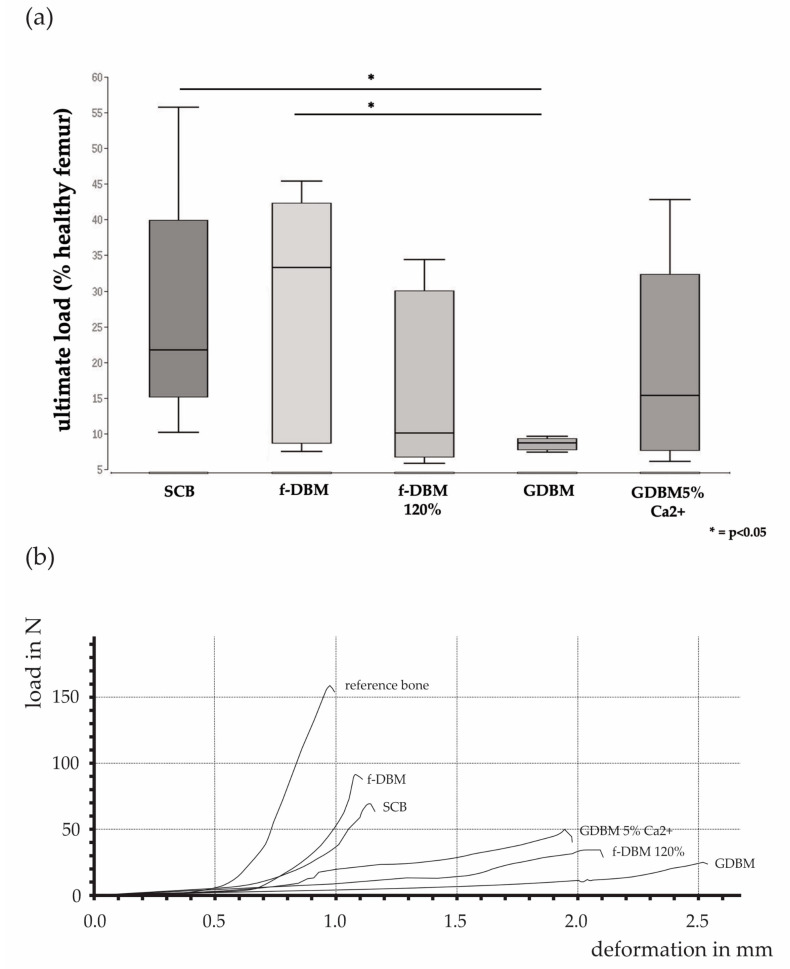
Ultimate load of the 5 mm bone defect, in relation to the contralateral healthy femur (= 100%). The percentage of ultimate load as percentage of the healthy contralateral femur was significantly lower in the GDBM group than in the SCB group and the f-DBM group. No significant differences between the fiber groups and no significant differences between the fiber groups and the SCB group (**a**). Selected force curves of the different groups show the deformation in mm per load in newtons (**b**).

The ultimate load as percentage of the healthy contralateral femur was observed to be significantly lower in the GDBM group (median: 8.7%) than in the SCB group (median: 21.8% vs. GDBM *p* < 0.05) and the f-DBM (median: 33.3% vs. GDBM *p* < 0.05) group. There were no significant differences between the fiber groups and especially no significant differences between the fiber groups and the SCB group. The highest median of all groups was observed in the f-DBM group (median: 33.3%) (Figure 2).

### 3.3. Similar Bone and Cartilage Formation in All Groups but Significantly Bigger Remnant Defect Size in GDBM

The analysis of histological slides stained with Movat pentachrome revealed no significant differences in bone or cartilage formation between the treatment groups.

**Figure 3:**
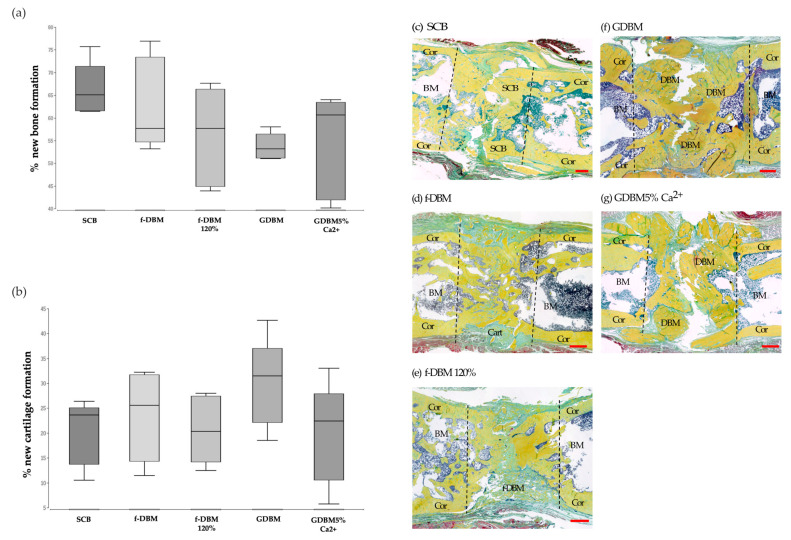
Percentage of new bone formation (**a**) and percentage of new cartilage formation (**b**). No significant differences between the groups. Movat pentachrome staining of SCB (**c**), f-DBM (**d**), f-DBM 120% (**e**), GDBM (**f**) and GDBM5%Ca2^+^ (**g**). Bone tissue appears yellow, cartilage greenish. Histology: scale bar represents 1 mm, BM = bone marrow, Cor = corticalis, Cart = cartilage.

Regarding new bone formation in the defect, the highest median was found in the SCB group (median: 65.1%). Similar median values were also observed in the two fibrous groups (median f-DBM: 57.7%; median f-DBM 120%: 57.6%). The lowest value was found in the GDBM group (median: 53.15%). Differences between the groups were without statistical significance. Cartilage formation was most prominent in the GDBM group (median: 31.5%) (Figure 3).

### 3.4. High Bone Healing Scores in f-DBM and Syngenic Cancellous Bone Groups

In general, the values of the calculated bone healing scores were closely related, as the previous histological evaluation already suggested, and no significant differences between the groups were observed. Nevertheless, the highest bone healing scores were found in the f-DBM and SCB groups with 25 points. The lowest score was found in the GDBM 5% calcium phosphate group with 20 points (Table 2).

### 3.5. Bone Mineral Density Significantly Higher in SCB Group Than Fiber Groups

**Figure 4:**
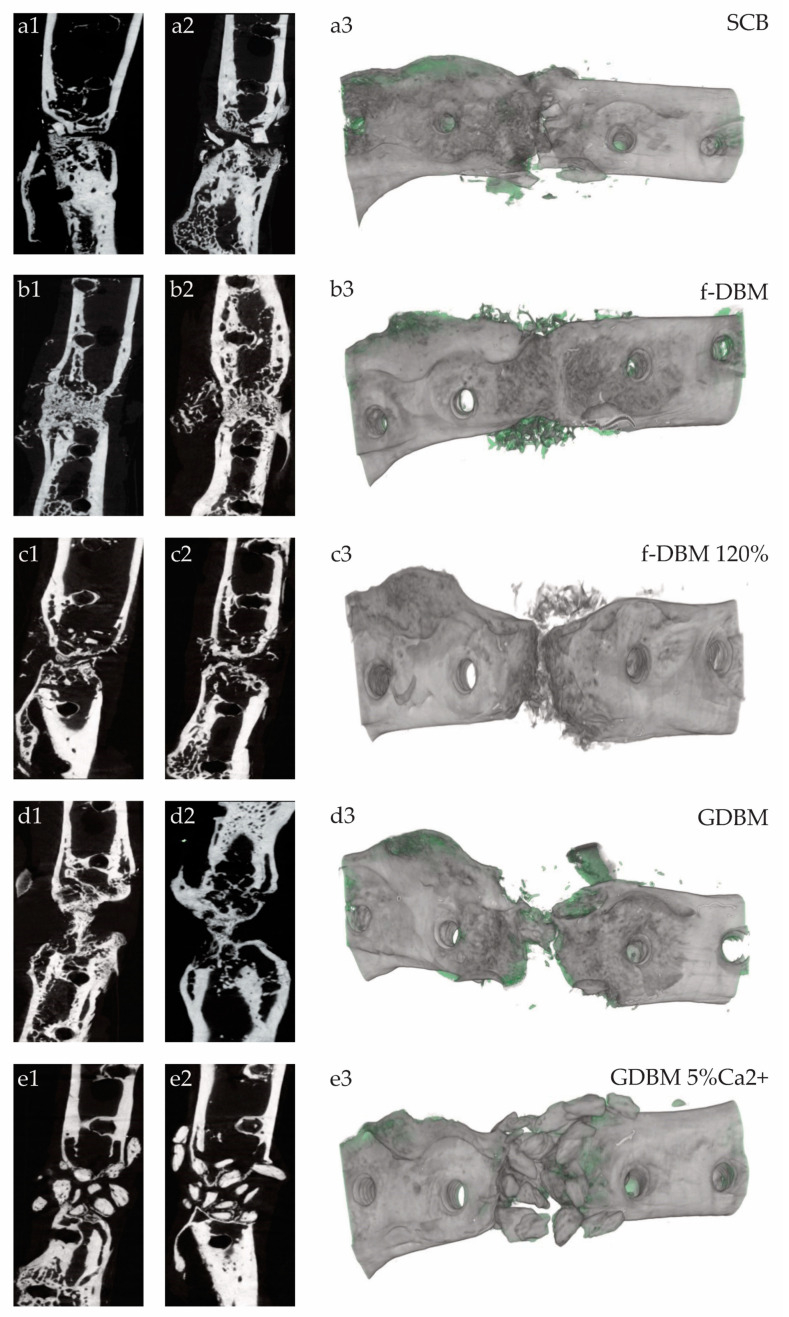

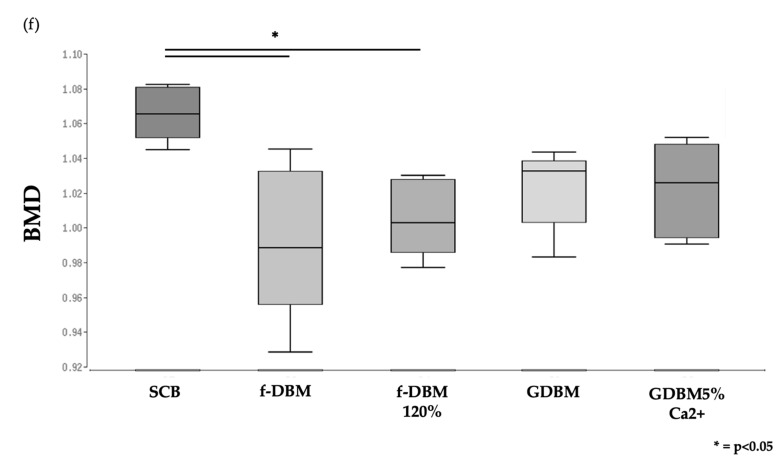
Bone healing results after 8 weeks in the different groups (**a**–**e**) in µCT slices (1–2) and 3D reconstructed (3). (**a**): SCB with high degree of integration. (**b**): f-DBM showing multiple bony bridges and almost no remnant defect size. (**c**): f-DBM 120% with bigger remnant defect size. (**d**): GDBM with remaining defect size and remaining granules. (**e**): GDBM5%Ca2^+^ with almost no osseous integration of the scaffolds. Bone mineral density (BMD) of the different groups (**f**). Significantly higher BMD in SCB group than in f-DBM and f-DBM 120% (* = *p* < 0.05).

In the µCT examinations performed, the f-DBM group (Figure 4b1–3) showed respectable bone remodeling and bone formation; continued bone consolidation was observed in the SCB (Figure 4a1–3) and f-DBM 120% (c1–3) groups. In the GDBM group (Figure 4d1–3), granule integration into bone tissue was extensively detectable, whereas in the GDBM5%Ca2^+^ group (Figure 4e1–3), granule integration was reduced.

The bone mineral density (BMD) in the defect area was significantly lower in the fiber groups (median f-DBM 0.9883, median f-DBM 120% 1.007) (*p* > 0.05) in comparison to the SCB group (median: 1.0653). The GDBM and GDBM5%Ca^2+^ groups showed no significant alteration in BMD compared to the SCB group (GDBM 1.0327, GDBM5%Ca^2+^ 1.0258) (Figure 4f). Bone volume to total volume ratio (BV/TV) was measured and calculated. The highest BV/TV was measured in the fDBM 120% group, followed by SCB and fDBM. The lowest value was detected in the GDBM group (Table 3).

### 3.6. Significantly Better Vascularization in GDBM Group Than GDBM with Calcium Group

**Figure 5:**
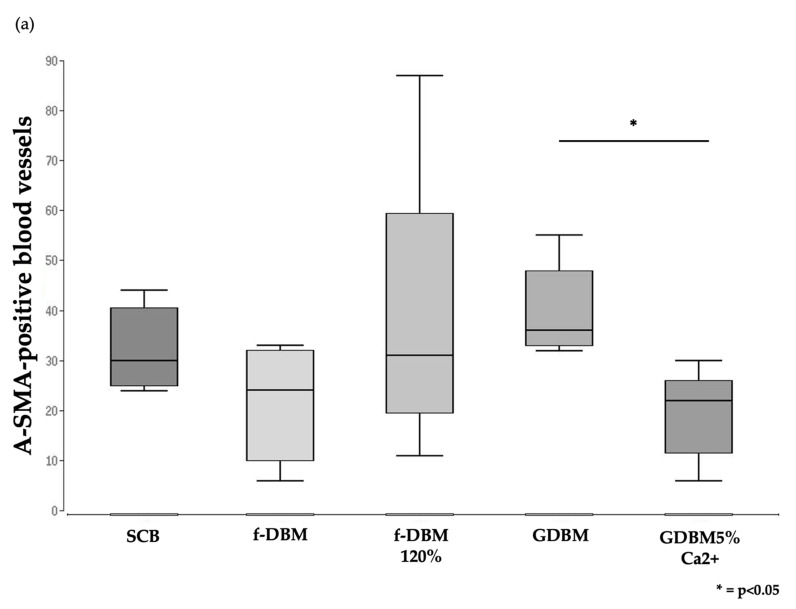

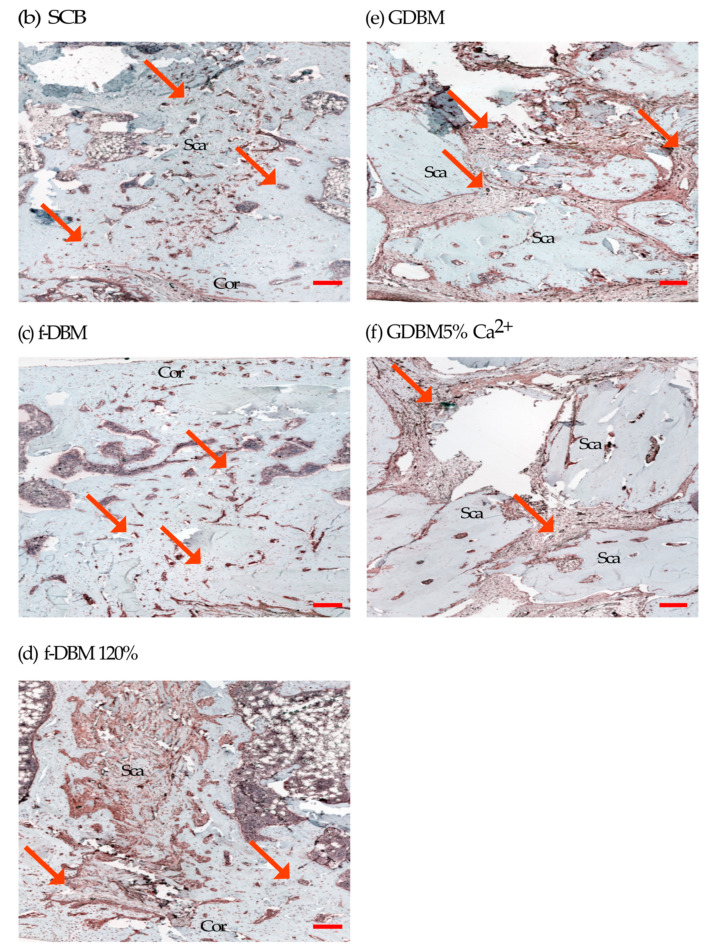
Histologically visible number of α-SMA-positive vessels within the defect area (**a**). Significantly fewer α-SMA-positive vessels were found in the GDBM5%Ca2^+^ group than in GDBM granules. Evaluation of vascularization based on immunostaining of established blood vessels, using antibodies directed against α-SMA within the whole defect. Representative images of bone defects treated with different scaffolds (**b**–**f**). α-SMA-positive vessels were more inhomogeneously distributed with a concentration in the peripheral area in the fDMB 120% group. Red arrows indicate α-SMA-positive blood vessels, Cor = corticalis, sca = scaffold. Scale bar indicates a distance of 200 μm; * *p* < 0.05.

A significantly better vascularization was observed in defects filled with GDBM (median: 36.0 vessels per field of view (vfv)) compared to defects implanted with GDBM5%Ca2^+^ (median: 22.0 vfv, *p* < 0.05), whereas only minor differences were found between the other groups (f-DBM 120%: median 31.0 vfw, SCB: median 30.0 vfw, Figure 5). The tighter packing of the fibers in the f-DBM 120% group did not result in a decrease in vessel density in the defect area but did result in a more inhomogeneous distribution of vessels with a concentration in the peripheral area (Figure 5).

## 4. Discussion

In this study, the effects of new DBM application forms combined with BMCs on the bone healing in a critically sized femoral defect of the rat were investigated and compared to the gold standard treatment. The different DBM application forms were transplanted after addition of BMCs into a plate-stabilized, femoral critical-size bone defect of 5 mm in SD rats. It was demonstrated that the newly developed fibrous application form with additional BMCs appears to be equivalent to the current gold standard syngenic cancellous bone in terms of bone healing. Tighter packing of the fiber material in the bone defect or a higher amount of residual calcium phosphate within the DBM did not improve bone healing in our experiments.

### 4.1. DBM in Bone Defect Treatment

Various DBM application forms are currently in clinical use. DBM products are used in the form of granules, sponges, strips, injectable putty, paste and paste supplemented with bone chips [37].

However, for their application, not only the physical properties of DBM products and the type of application (e.g., as a single substance, as part of a composite or as a cell carrier) but also the size and nature of the bone defect must be considered [38,39]. The best healing results were achieved in defects with close contact to vital bone and in small, well-vascularized defects [40,41]. All these conditions are found, for example, in the clearly defined dental alveolar compartment. At this location, DBM paste shows significantly better bone formation than synthetic bone replacement materials [42]. These ideal osteoconductive conditions can also be found in the field of spinal surgery in the freshly dissected intervertebral space for vertebral body fusion [43,44,45]. If one wants to examine DBM preparations for bone defect healing, it should be noted that these ideal conditions for DBM are restricted to certain anatomical sites.

Especially in the area of long tubular bones, care must be taken to ensure that the DBM remains within the bone defect; otherwise, bone healing may be reduced and/or undesirable ectopic bone healing may be induced.

Therefore, it is important to choose the defect model that is closest to the real situation. Oftentimes, the punch defect model in the rat skull is used as an analog to a critical-size defect [21,46,47]. Similar to the dental alveolar compartment, this defect provides a closed compartment to keep the implanted DBM in place, but the conditions are not comparable to a critical defect in a long tubular bone, which offers a significant mechanical stimulus contrary to bone defects of the skull and is vascularized.

This can best be represented in an animal model by a femoral defect [13,20,21,48], but due to the anatomical conditions, the closed compartment as in the punch defect does not exist. It is therefore necessary to provide a material form that is able to handle the mechanical load due to sufficient bone healing and remains at the location of the defect. All of these criteria are met with f-DBM. Bone defects treated with f-DBM show similar biomechanical properties to bone defects implanted with the current syngenic cancellous bone gold standard (Figure 2). Moreover, radiographic and histological examination shows satisfactory bone healing, extensive integration of the material to the host bone and newly formed bone (Figure 4a–e) (Table 3) and retention of the material at the site of the defect.

### 4.2. Tighter Packing and Higher Concentration of Residual Calcium Phosphate Does Not Improve Bone Healing

Studies on the amount and density of bone substitute material to be used are rare. In order to provide even better biomechanical stability due to bone healing, an attempt was made to increase the amount of DBM transplanted into the defect. In comparison to the normal defect filling, 20% more material was used. However, in our experiments, this led to neither an improvement in bone healing nor a decrease in newly formed vessels. Nevertheless, a more inhomogeneous distribution of vessels with a concentration in the peripheral area was observed in bone defects filled with densely packed fDBM. One of the reasons for poorer bone healing can be the lack of oxygen and nutrients in the defect center caused by the pressure which might inhibit the ingrowth of blood vessels. The lack of vascularization in turn leads to a perpetuation of hypoxic conditions inside the graft, which ultimately leads to the manifestation of avital areas that do not contribute to the formation of bone tissue but to the destabilization of the defect area. Conversely, various studies have shown that bone healing in the defect area is oxygen-dependent [49,50]. In addition, it can be assumed that the denser packing will prevent the applied BMCs from developing their full potential due to the lack of nutrient and oxygen supply.

The demineralization of the bone matrix during the production process of DBM and its derivates leads to a lack of osteoinductive ions such as calcium. Calcium ions can induce the expression of the bone growth factor BMP-2 [25,26,27] and enhance differentiation and survival of cells as well as induce early vascularization in critically sized bone defects [28,29]. However, with a residual calcium phosphate content of 5% in the G-DBM, neither a stimulation of vascularization nor an improvement of bone healing could be observed compared to completely demineralized G-DBM. This finding could mean that a residual calcium phosphate content of 5% is too low to cause significant effects on bone tissue formation.

In fact, G-DBM+5% residual calcium phosphate led to a significant reduction in blood vessel density compared to G-DBM. It should be noted that calcium is usually present in the bone as calcium phosphate. It can only be speculated whether and how the increased residual calcium phosphate mediates the significant decrease in blood vessel density in the defect area after 8 weeks of healing. As described above, calcium ions also support proangiogenic processes, but the effect is dose-dependent [9,28,29]. In this respect, calcium phosphate release kinetics through the material could be relevant. Furthermore, the transition from calcium phosphate to ionic calcium and phosphate from the solid phase to the liquid phase seems to be important. Whether and how these products positively or negatively influence angiogenesis in vivo is not yet known to our knowledge. There is still a great need for research on this, as also noted by Malhotra and Habibovic in a review article [51].

### 4.3. Use of BMCs with f-DBM Leads to Gold-Standard-Like Bone Healing Results

Based on experimental results showing an improved osteogenic effect of the DBM through different types of biofunctionalization, e.g., by coating with BMP-2 [39] or BMCs [19], the different DBM forms in the present study were colonized with syngenic BMCs before transplantation into the defect. BMCs represent a heterogeneous mixture of cells that can support bone defect healing in different ways. Thus, using appropriate depletion experiments, it has been described in our preliminary work that the monocyte fraction in BMCs has a major role in defect healing [52], and a possible influence on vascularization was not measurable after 8 weeks of healing. The mean percentage of monocytes in BMCs is 13.8%. CD271-positive MSCs are another cell population in BMCs with a well-described osteoanabolic effect, but their proportion is much lower in BMCs at 0.06% [11,52]. However, their specific role in BMC preparations for bone healing has not yet been analyzed. One cell type in BMCs with a putative proangiogenic effect can be found in the fraction of CD34-positive hematopoietic stem cells [53] that were defined as endothelial progenitor cells (EPCs). The CD34+ fraction in BMCs is on average 3–4% [11,52]. However, its supportive effect in BMCs for bone healing and vascularization remains questionable; depletion experiments showed no significant influence on both parameters after 8 weeks [52].

## 5. Conclusions

The application of DBM in fiber form combined with the application of BMCs shows promising results in animal experiments regarding bone healing. Results comparable to the gold standard syngenic cancellous bone have been achieved. Denser packing of fibers or a higher amount of calcium phosphate does not seem to have a positive effect. However, further studies are needed to evaluate the exact mechanistic aspects.

## Figures and Tables

**Table 1:** Groups of different material combinations and number of animals assigned to evaluation group.

Material	Histology	Radiology/Biomechanical Testing
syngenic cancellous bone (SCB)	*n* = 5	*n* = 8
fibrous demineralized bone matrix (f-DBM)	*n* = 5	*n* = 8
fibrous demineralized bone matrix densely packed (f-DBM 120%)	*n* = 5	*n* = 8
DBM granules (GDBM)	*n* = 5	*n* = 8
DBM granules 5% calcium phosphate (GDBM5%Ca^2+^)	*n* = 5	*n* = 8

**Table 2:** Calculated bone healing scores in the different groups.

Material.	Bone Healing Score
syngenic cancellous bone (SCB)	25
fibrous demineralized bone matrix (f-DBM)	25
fibrous demineralized bone matrix densely packed (f-DBM 120%)	22
DBM granules (GDBM)	23
DBM granules 5% calcium phosphate (GDBM5%Ca2^+^)	20

**Table 3:** Bone volume and bone volume/total volume in the different groups.

Material	Bone Volume/Total Volume (BV/TV)
syngenic cancellous bone (SCB)	0.8530 (±5.3%)
fibrous demineralized bone matrix (f-DBM)	0.8303 (±4.8%)
fibrous demineralized bone matrix densely packed (f-DBM 120%)	0.8803 (±5.5%)
DBM granules (GDBM)	0.7514 (±5.9%)
DBM granules 5% calcium phosphate (GDBM5%Ca2^+^)	0.7988 (±4.4%)

## Data Availability

The data presented in this study are available on request from the corresponding author.
